# Effects of Test-Section Length and Cross-Sectional Blockage on the Swimming Performance of Juvenile Grass Carp: Implications for Standardized Testing and Fish-Passage Design

**DOI:** 10.3390/ani16172710

**Published:** 2026-09-01

**Authors:** Jianzhang Lv, Jingjuan Li, Zixuan Li, Biao Wang, Yinzhu Liu, Huijuan Chen, Xiaogang Wang

**Affiliations:** 1 Hydraulic Engineering Department, Nanjing Hydraulic Research Institute, Nanjing 210029, China; jzlv@nhri.cn (J.L.); jjli@nhri.cn (J.L.); biaowang@hhu.edu.cn (B.W.); 2 State Key Laboratory of Hydraulic Engineering Intelligent Construction and Operation, Tianjin University, Tianjin 300350, China; 3 Jiangdu Key Water Conservancy Project, Yangzhou 225200, China; 4 School of Marine Science and Fisheries, Jiangsu Ocean University, Lianyungang 222005, China

**Keywords:** juvenile grass carp, critical swimming speed, test-section length, cross-sectional blockage ratio, spatial constraint, swimming behavior, fish passage

## Abstract

Closed-flume spatial constraints may bias swimming-performance data used in fish-passage design. Two independent experiments were conducted using different size classes of juvenile grass carp (*Ctenopharyngodon idella*). Fish with a mean body length (BL) of 7.92 ± 0.61 cm were used to assess five test-section lengths (8, 6, 4, 3.5, and 3 BL), whereas fish with a mean BL of 15.76 ± 0.97 cm were used to assess three blockage ratios (1.88%, 9.26%, and 15.31%). Incremental-velocity tests quantified critical swimming speed and behavior, and three-dimensional simulations examined the local flow field under the 9.26% blockage. Relative critical swimming speed was similar at 4–8 BL (9.71–9.95 BL/s) but decreased by 10.96% from 4 to 3.5 BL. Increasing blockage from 1.88% to 9.26% and 15.31% reduced it from 6.99 to 5.34 and 5.24 BL/s, respectively. Behavioral responses also depended on spatial constraint: shorter sections reduced the space available for forward movement and postural adjustment, whereas higher blockage restricted lateral body and tail movements. As velocity increased, fish intensified tail beating; under higher blockage, compensation relied increasingly on tail-beat frequency because amplitude adjustment was constrained, with greater variation among individuals. Simulations showed that local streamwise velocity near the maximum fish cross-section was 20.8–25.0% above inlet velocity. At least 4 BL is recommended for fish comparable to those in the length experiment. Below 5% is suggested as a precautionary blockage target.

## Simple Summary

Reliable measurements of how well fish swim are needed to design passages that help fish move past dams and other barriers. However, the size of the testing chamber may change the measured result. We tested young grass carp in chambers of different lengths and widths and also examined how water moved around the fish. Short chambers reduced the distance available for the fish to accelerate and regain position. Narrow chambers made water flow faster around the body and reduced the measured swimming speed. The results indicate that test chambers should provide sufficient space for fish movement and minimize constraints imposed by the chamber boundaries whenever possible. These findings can help laboratories obtain more comparable swimming measurements and can improve the use of those measurements in fish-passage design.

## 1. Introduction

Fishways are widely used to mitigate the loss of longitudinal connectivity caused by dams and weirs, and their effectiveness depends on matching hydraulic conditions to the swimming capacity of target fish [1]. Swimming-performance metrics, including induced velocity, critical swimming speed, and burst swimming speed, together with observations of swimming behavior, are commonly related to fishway hydraulic conditions to evaluate attraction and passage suitability and guide fishway design and operation [2,3,4]. Accurate characterization of swimming performance and behavioral responses is therefore fundamental to understanding fish locomotor capacity and supporting biologically informed fishway design.

Measured fish swimming performance is jointly affected by fish traits, external environmental conditions, and testing methods. Critical swimming speed is currently one of the most widely used indices of swimming performance and can comprehensively reflect sustained swimming capacity under stepwise increases in flow velocity. In a synthesis of 204 freshwater fish studies, Cano-Barbacil et al. [5] reported that body length, water temperature, body morphology, velocity increment, and increment duration can significantly affect critical swimming speed. Jones et al. [6] found clear variation in swimming performance among species, populations, and individuals, and showed that the relationship between body length and swimming speed differs among species. In addition, water temperature, velocity, and turbulence jointly affect swimming performance [7], while body morphology and propulsion mode can alter locomotor energy costs in unsteady flows [8]. However, critical swimming speed mainly reflects the integrated capacity of a test fish before fatigue and cannot independently describe changes in propulsion mode, posture control, or spatial position as flow velocity increases. Kraskura et al. [9] noted that maximum anaerobic swimming capacities, such as burst and leaping performance, have received relatively limited attention in existing studies of swimming performance. Ashraf et al. [10] further analyzed burst swimming capacity in relation to swimming speed and body length from the perspective of time to exhaustion. Kinematic studies have shown that fish can adjust tail-beat frequency, body undulation, and spatial position according to incoming-flow structure, and different flow fields at the same mean velocity may correspond to different locomotor adjustments and energy costs [11]. Therefore, jointly analyzing tail-beat motion, burst behavior, and the time budget of swimming states with critical swimming speed can help identify locomotor adjustments during velocity increases and explain the behavioral sources of differences in measured critical swimming speed under different test conditions.

In addition to fish traits and environmental conditions, testing protocols and experimental apparatus also influence measurements of swimming performance. Bamford and Seebacher [12] found that rapid critical swimming-speed tests had individual repeatability similar to that of conventional tests, but differed in test duration, recovery requirements, and repeated-testing effects. Illing et al. [13] improved flow uniformity and test repeatability by optimizing velocity control and test-section structure in a multi-lane swimming chamber, while also finding that different fish species did not respond consistently to the same protocol. Ashraf et al. [14] further reported an interaction between flume length and fatigue criteria, indicating that different fatigue definitions may alter assessments of flume-length effects. Beyond test protocols, the way velocity is characterized within the test section is also important for accurately calculating swimming performance. Combining flume experiments and numerical simulations, Vezza et al. [15] found that, for bottom-swimming European glass eel, using cross-sectional mean velocity instead of near-bed velocity at the fish position overestimated swimming performance by an average of 18–32%; distinguishing active swimming time from gliding and drifting time produced results that more accurately reflected the actual locomotor state of the test fish. More recently, hyperbaric swimming respirometers and adjustable sprint chambers have been used to simulate different depth environments and measure multiple sprint traits [16,17], further indicating that testing apparatus should be matched to the target swimming-performance index and the corresponding mode of locomotion. Thus, in addition to testing protocols and fatigue criteria, the internal flow field of the test section and the movement space it provides are important conditions affecting measured swimming performance.

The spatial scale of a test section mainly includes longitudinal length and cross-sectional scale. Longitudinal length determines the distance available for sprint acceleration, longitudinal displacement, and recovery of position after downstream retreat. Cross-sectional scale may influence the proportion of the flow area occupied by the fish body and the space available for lateral movement, vertical adjustment, and tail-beat motion. Cross-sectional blockage ratio is usually defined as the ratio of the maximum cross-sectional area of the fish body to the flow cross-sectional area of the test section. As the blockage ratio increases, the effective flow area between the fish body and the sidewalls decreases, which may induce local bypass-flow acceleration while compressing the space available for tail beating and postural adjustment. Previous studies have shown that changing test-section length may affect evaluations of fish swimming performance [14], and that wall form and the local hydrodynamic conditions it creates can alter swimming and station-holding behavior in juvenile common carp [18]. Govindasamy et al. [19] found that, under their specific bottom-roughness and vertical-position conditions, bottom swimming did not significantly reduce the hydrodynamic forces experienced by round goby, indicating that fish use of near-wall flows and the associated hydrodynamic effects are jointly influenced by species and boundary conditions. Grass carp is an important species in major inland river systems in China and is a common target species in fish passage design and research for inland rivers. For juvenile grass carp, Cao et al. [20] coupled upstream trajectories with the flow field of a vertical-slot fishway and found that test fish mainly selected regions with velocities of 0.01–0.45 m/s and avoided regions where turbulent kinetic energy exceeded 0.012 m^2^/s^2^. Related reviews also indicate that rheotactic response, spatial use, and locomotor behavior of grass carp vary with life stage and external environment [21]. Overall, existing studies have examined the effects of apparatus conditions on fish swimming performance from the perspectives of test-section length, wall conditions, local velocity, and near-wall swimming.

However, most existing studies have relied primarily on critical swimming speed or time to exhaustion, with limited consideration of associated changes in tail-beat motion, burst swimming, and positional regulation. Two related questions therefore remain unresolved, particularly for juvenile grass carp. First, it remains unclear whether critical swimming-speed measurements remain stable across different test-section lengths or show a transition as longitudinal space is reduced. Second, how cross-sectional blockage affects swimming performance through the combined influence of local flow-field modification and reduced movement space remains insufficiently understood. To address these gaps, this study used a closed swimming flume with an adjustable test-section spatial scale to conduct two independent experiments on test-section length and cross-sectional blockage ratio. Test-section lengths were set to 3, 3.5, 4, 6, and 8 body lengths (BL), and cross-sectional blockage ratios were set to 1.88%, 9.26%, and 15.31%. Absolute and relative critical swimming speeds of juvenile grass carp were measured, while tail-beat amplitude, tail-beat frequency, burst frequency, burst speed, and the time proportions of different swimming states were recorded simultaneously. These data were used to systematically analyze the effects of longitudinal-scale constraint and cross-sectional blockage on swimming performance and behavioral responses. In addition, a typical condition with a cross-sectional blockage ratio of 9.26% was selected to establish a three-dimensional numerical model. The model simulated the local velocity distribution and bypass-flow characteristics when a straight-bodied fish held station at the center of the test section, thereby helping to explain the hydrodynamic mechanism through which cross-sectional spatial constraint affects critical swimming speed and station-holding behavior. This study aimed to reveal how test-section length and cross-sectional blockage ratio affect swimming-performance measurements in juvenile grass carp and to identify suitable ranges for these parameters, providing a basis for scale design of swimming performance test apparatus, standardization of experimental conditions, and comparison of data among different devices, and ultimately improving the reliability of biological parameters required for fish passage design.

## 2. Materials and Methods

### 2.1. Experimental Apparatus and Test Conditions

**Figure 1:**
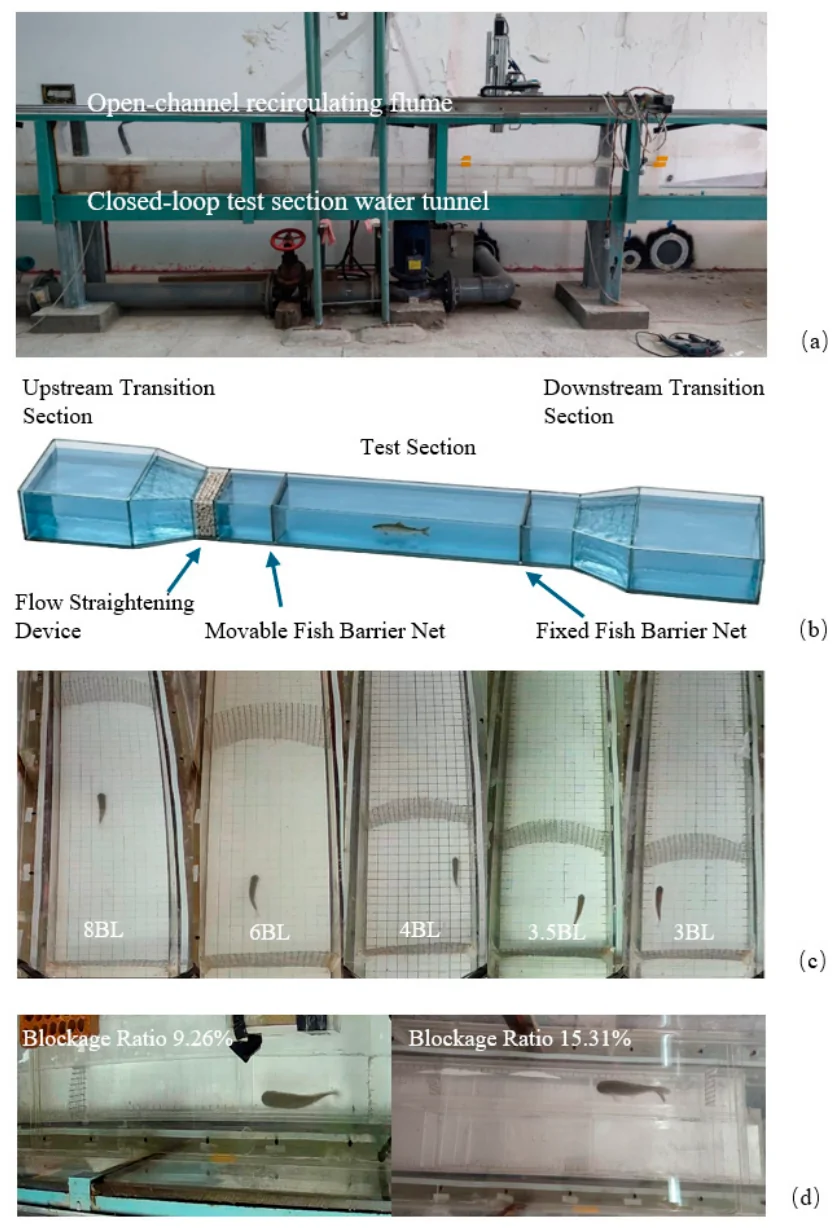
Experimental apparatus and test-condition settings: (**a**) recirculating open-channel flume and closed test section; (**b**) schematic of the closed test section; (**c**) test-section length conditions; (**d**) cross-sectional blockage-ratio conditions.

Experiments were conducted in a closed flume installed within a recirculating open-channel flume. The open-channel flume was 600 cm long, 30 cm wide, and 35 cm high. Transparent acrylic plates were added to create a closed test flow field that met the experimental requirements, and the top cover of the closed flume was removable, as shown in Figure 1a,b. A camera was mounted directly above the open-channel flume to record the entire swimming experiment of juvenile grass carp for subsequent analysis of swimming behavior and state. Upstream of the closed test section, a gradually contracting bell-mouth inlet connected the incoming flow from the open-channel flume and was equipped with a flow-straightening device. The straight section of the closed flume contained a movable fish screen and a fixed fish screen located 300 cm downstream of the upstream flow straightener; the region between the two screens served as the experimental area. Because the movable screen was positioned far from the flow straightener, the test-section flow could fully develop and maintain relatively low turbulence, producing a more uniform flow field than that in an annular flume. Downstream of the fixed screen, a gradually expanding bell-mouth outlet connected smoothly with the open-channel flume. Before the experiments, the relationship between pump discharge and test-section velocity was calibrated separately for each cross-sectional condition following the general procedure of Song and Xie [22]. Discharge was recorded using an electronic flow meter installed in the inlet pipeline. After the flow stabilized at each discharge setting, velocity was measured with a propeller current meter at the upper, middle, and lower positions along the vertical centerline at the middle of the test section. The mean of the three measurements was taken as the inlet velocity. Linear calibration produced slopes of 0.69, 3.26, and 5.70 cm/s per m^3^/h for the 20 cm × 20 cm, 9 cm × 9 cm, and 7 cm × 7 cm test sections, respectively, with all *R*^2^ values exceeding 0.98.

Five test-section-length treatments were conducted by varying test-section length. When the cross-sectional dimensions of the test section were 20 cm × 20 cm, the following test-section length conditions were set by adjusting the movable fish screen based on the mean body length of the test fish: 8 BL, 6 BL, 4 BL, 3.5 BL, and 3 BL, as shown in Figure 1c.

Three blockage-ratio treatments were conducted by varying the cross-sectional area of the test section. When the test-section length was 6 BL, the cross-sectional area was changed by adding L-shaped blocks to the closed-flume test section. Based on the cross-sectional area of the experimental fish (maximum body cross-sectional area approximately 7.5 cm^2^), three blockage-ratio conditions were established: the 1.88% group, with test-section dimensions of 20 cm × 20 cm; the 9.26% group, near the commonly used empirical threshold for blockage ratio, with test-section dimensions of 9 cm × 9 cm; and the 15.31% group, with test-section dimensions of 7 cm × 7 cm, as shown in Figure 1d. Because body size varied slightly among individuals, the blockage-ratio treatments were defined using nominal rather than individual-specific values. A representative maximum fish cross-sectional area of approximately 7.5 cm^2^ was estimated by approximating the body cross-section as an ellipse, and the nominal blockage ratios were calculated relative to the fixed cross-sectional area of each test section. Treatment assignment and subsequent analyses were based on the nominal blockage ratios of 1.88%, 9.26%, and 15.31%.

Compared with conventional annular swimming flumes, the present apparatus uses a straight closed test section preceded by flow-straightening and flow-development sections, thereby reducing the influence of channel curvature and secondary flow on the test-area flow field. In addition, the longitudinal movement space and cross-sectional blockage ratio can be independently adjusted by changing the position of the movable fish screen and the cross-sectional dimensions of the test section, making the apparatus particularly suitable for examining fish swimming performance and behavioral responses under different spatial constraints.

### 2.2. Test Fish

Healthy and active juvenile grass carp were selected as the study animals. After arrival at the laboratory, all test fish were acclimated for 2 weeks in indoor rectangular tanks. The acclimation water was dechlorinated tap water that had been continuously aerated for more than 5 d. Water temperature was maintained at 25 ± 1 °C, and dissolved oxygen was kept above 7.50 mg/L through continuous aeration. To reduce the influence of specific dynamic action on aerobic metabolism, all test fish were fasted for 48 h before formal testing. For each experiment, fish from the corresponding size class were netted individually from the holding tank without deliberate selection and allocated sequentially among treatments until the required sample size was reached.

For the test-section length experiment, juvenile grass carp with a mean body length of 7.92 ± 0.61 cm were used. Their maximum cross-sectional area was approximately 1.7 cm^2^, and their morphological parameters are shown in Table 1. Under the fixed cross-sectional condition of 20 cm × 20 cm, the fish body cross-sectional blockage ratio was approximately 0.43%, which minimized interference from cross-sectional blockage in the analysis of test-section length effects. For the cross-sectional blockage-ratio experiment, juvenile grass carp with a mean body length of 15.76 ± 0.97 cm were used. Their maximum cross-sectional area was approximately 7.5 cm^2^, and the relative test-section length was fixed at 6 BL to focus on the influence of cross-sectional spatial constriction. The morphological parameters of these fish are shown in Table 2. Because the two experiments used different fish-size groups, they were treated as independent assessments, and comparisons were made only among treatments within each experiment.

The two experiments used juvenile grass carp of different sizes as independent designs based on test-section geometry, target blockage-ratio range, and single-factor control requirements. If the smaller fish used in the test-section length experiment had also been used in the blockage-ratio experiment, the side lengths of the square test section would have needed to be reduced to approximately 4.3 and 3.3 cm to generate blockage ratios of 9.26% and 15.31%, respectively. Such small cross-sectional dimensions would make it difficult to ensure sufficient flow development, allow postural adjustment, record swimming behavior, and operate the apparatus. Therefore, larger juvenile grass carp were used for the blockage-ratio experiment, and test sections of 9 cm × 9 cm and 7 cm × 7 cm were constructed to produce the corresponding blockage ratios. Subsequent statistical analyses and result comparisons were conducted separately within each experiment.

### 2.3. Measurement of Critical Swimming Speed

Critical swimming speed was tested using the incremental-velocity method proposed by Brett, combined with the specification for testing swimming ability of target fishes for hydropower projects [23,24]. Before testing, a single test fish was placed in the test section for acclimation at a flow velocity of 1 BL/s for 1 h. The initial estimated critical swimming speed for each experimental size class was calculated from its mean body length using the empirical relationship for juvenile grass carp reported by Gong et al. [25]:

(1) $$U_{\mathrm{crit},\mathrm{est}}=3.96\cdot \mathrm{BL}+47.49$$

where *U*_crit,est_ is the estimated critical swimming speed (cm/s) and BL is the mean body length (cm). This estimate was used only to determine the transition to the formal velocity-increment stage. After the test began, flow velocity was increased by 0.5 BL/s every 5 min until 60% of the estimated critical swimming speed was reached. The velocity increment was then adjusted to 0.8 BL/s, with a velocity-step interval of 15 min. A fish was considered exhausted when it remained in continuous contact with the downstream screen for 20 s and did not resume swimming. This duration followed previously published critical swimming-speed protocols and was used to distinguish sustained loss of station-holding ability from brief, recoverable contact with the screen [26]. The swimming time and velocity at the highest velocity step were recorded as the test result, and the trial was terminated. Critical swimming speed, *U*_crit_, was calculated using Equation (2):

(2) $$U_{\mathrm{crit}}=U_{\max}+\Delta U\frac{t_{f}}{\Delta t}$$

where Δ*U* is the prescribed velocity increment (cm/s), Δ*t* is the velocity-step duration (min), *U*_max_ is the maximum swimming velocity that the test fish could complete for the full duration (cm/s), and *t*_f_ is the swimming time of the test fish at the highest velocity step (min).

### 2.4. Swimming Behavior Methods

During testing, a high-definition camera fixed directly above the flume recorded the movement trajectories and swimming behavior of the test fish from a top-view perspective throughout the trial. Video recordings were imported into Logger Pro software (version 3.8.6.1; Vernier Software & Technology, Beaverton, OR, USA) for frame-by-frame tracking and quantitative kinematic analysis. Because the recordings provided only a top view, quantitative analysis was restricted to variables that could be reliably identified in the horizontal plane, including body heading, longitudinal displacement, lateral tail-beat amplitude, and tail-beat frequency; vertical position, pitch, and roll were not quantified.

Tail-beat parameters were extracted following the methods of Bainbridge and Webb et al. on the relationships among swimming speed, tail-beat frequency, and tail-beat amplitude [27,28]. The classification of swimming states and locomotor behavior followed studies on gait transitions and swimming-behavior categories in fish [29,30], with additional judgment based on fish orientation relative to the flow direction, displacement direction, and postural changes observed in the videos. To examine swimming-strategy responses of juvenile grass carp under different apparatus dimensions, the following kinematic and behavioral indices were analyzed:

(1) Tail-beat amplitude (TBA): the peak-to-peak lateral excursion of the caudal-fin tip during one complete tail-beat cycle. The position of the caudal-fin tip was marked frame by frame in Logger Pro, and the two frames corresponding to its extreme lateral positions on opposite sides were identified. The lateral distance between these two positions was taken as TBA and normalized by the body length (BL) of the individual fish.
(2) Tail-beat frequency (TBF): the number of complete caudal-fin oscillation cycles per unit time. To ensure that the statistical data represented a relatively steady state, the mean tail-beat frequency was calculated continuously over the following 5 min after each velocity step had run for 6 min.
(3) Burst frequency: a single burst was defined as explosive swimming that produced an upstream relative displacement greater than 1 BL through rapid tail beating. The frequency of bursts at each velocity step was recorded in events per minute. To reduce the influence of transient behavioral disturbance immediately after velocity switching, only valid data from the 2nd to the 15th minute of each velocity step were included.
(4) Burst speed: the displacement and duration of a single burst were extracted from the onset of acceleration until the ground-relative velocity decreased to zero, and the mean relative burst speed was calculated in BL/s.
(5) Time budget of swimming states: swimming behavior was classified into four states according to fish orientation relative to the flow, body posture, and displacement characteristics: station holding upstream (SHU), forward swimming upstream (FSU), upstream-facing backward movement (BSU), and passive drifting downstream (PDD). SHU was defined as the fish maintaining an upstream-facing posture and continuous tail beating with longitudinal displacement less than 0.5 BL. FSU was defined as active displacement in the upstream direction. BSU was defined as downstream displacement while the fish maintained an upstream-facing posture. PDD was defined as downstream movement with the fish’s head not oriented toward the flume inlet—that is, the absolute angle between the fish’s heading direction and the upstream direction toward the inlet was greater than 90°. Tail-beat frequency was extremely low, and tail movements were used mainly to stabilize body orientation. The time proportion of each state during the formal testing stage was calculated. For each fish, the proportion of time spent in each swimming state was calculated separately. The percentages reported for each treatment and flow-velocity condition represent the mean individual proportions across all fish that reached and completed the corresponding analyzed velocity period.

Swimming-behavior analysis was performed only for the formal testing stage after 60% of the estimated critical swimming speed had been reached, when each velocity step lasted 15 min. The preceding rapid increment stage, in which each step lasted 5 min, was used only to gradually increase flow velocity and was not included in the statistics for tail-beat motion, burst behavior, or the time budget of swimming states.

### 2.5. Numerical Simulation of the Test-Section Flow Field

To further analyze the influence of cross-sectional blockage on the local flow field around juvenile grass carp, a typical condition corresponding to the 9.26% blockage-ratio experiment was selected to establish a three-dimensional numerical model. The cross-sectional dimensions of the test section were 9 cm × 9 cm. The computational domain was 6 BL in length. The juvenile grass carp model had a total length of 19 cm, with maximum body depth and body width of 3.8 and 2.5 cm, respectively, and its maximum cross-section was located approximately 7 cm from the snout. To emphasize the local blockage and bypass-flow effects generated by the fish body occupying the flow cross-section, and to control for the additional influence of changes in swimming posture and spatial position on the flow field, the juvenile grass carp was set as a straight, stationary body in the numerical simulation. Its longitudinal body axis coincided with the longitudinal centerline of the test section, and the fish was located at the geometric center of the test-section cross-section.

Numerical calculations were conducted using the finite volume method, with inlet velocities of 0.4, 0.8, and 1.2 m/s. A velocity-inlet boundary condition was applied at the test-section inlet, and a pressure-outlet boundary condition was applied at the outlet. The four walls of the test section and the fish-body surface were set as no-slip walls. The *k*-*ω* turbulence model was used to solve the flow around the fish body. The main computational domain was meshed automatically, and local refinement was applied near the fish-body surface, dorsal fin, and caudal fin to improve the resolution of near-wall velocity gradients and local bypass-flow characteristics. The mesh size on the fish-body surface was set to 0.004 m. The first near-wall mesh layer was arranged according to a target *y*^+^ ≈ 1, and the growth rate of the boundary-layer mesh was 1.1. A relatively coarser mesh was used in fluid regions far from the fish body to balance computational accuracy and efficiency. Following a mesh-independence test, a mesh containing approximately 7.0 million cells was adopted for the numerical simulations.

The numerical model was validated using point velocity measurements obtained under the same test-section dimensions, fish model, and incoming-flow conditions. The relative error between simulated and measured velocities was 1.5–9.5%, all below 10%, indicating that the model could adequately represent the local velocity distribution around the fish body and was suitable for subsequent flow-field analysis. After calculation, streamwise velocity distributions were extracted from the longitudinal vertical center plane and the longitudinal horizontal center plane to analyze flow separation in front of the fish, local acceleration near the maximum cross-section, and wake development downstream under different incoming-flow velocities.

### 2.6. Statistical Analysis

Experimental data are expressed as mean ± standard deviation. For absolute and relative critical swimming-speed data, the Shapiro–Wilk test and Levene’s test were used before statistical analysis to assess normality and homogeneity of variance, respectively. Because the assumptions of normality and/or homogeneity of variance were not satisfied, the Kruskal–Wallis nonparametric test was used to compare differences among test-section-length conditions or among cross-sectional blockage-ratio conditions, followed by Dunn–Bonferroni post hoc pairwise comparisons. Individual fish were treated as independent statistical units for critical swimming-speed analysis. Overall effect sizes were quantified using epsilon squared (*ε*^2^), and 95% confidence intervals for the reported between-group mean differences were estimated using the bias-corrected and accelerated bootstrap method with 100,000 resamples. All tests were two-sided, and the significance level was set at *p* < 0.05. Different lowercase letters in the figures indicate significant differences among groups, whereas identical letters indicate no significant difference. Tail-beat amplitude, tail-beat frequency, burst speed, burst frequency, and the time proportions of different swimming states were analyzed mainly using descriptive statistics.

For an illustrative comparison with the conventional solid-blockage estimate, the Bell–Terhune correction was calculated as:

(3) $$U_{\mathrm{BT}}=U_{m}(1+\varepsilon_{s})$$

(4) $$\varepsilon_{s}=\tau \lambda {\left(\frac{A_{f}}{A_{t}}\right)}^{1.5}$$

where *U*_BT_ is the velocity after conventional solid-blockage correction, *U*_m_ is the measured velocity, *ε*_s_ is the fractional solid-blockage correction, *τ* is the tunnel cross-sectional shape coefficient (0.8), *λ* is the fish-body shape factor calculated as 0.5*L*/*T*, *L* is fish body length, *T* is maximum body thickness, *A*_f_ is the maximum fish cross-sectional area, and *A*_t_ is the cross-sectional area of the test section [31,32]. Group-mean morphological parameters in Table 2 and the nominal test-section dimensions were used in this illustrative calculation. The corrected values were not used in statistical testing or as replacements for the measured *U*_crit_ values.

## 3. Results

### 3.1. Effects of Test-Section Length on Critical Swimming Speed and Swimming Behavior of Juvenile Grass Carp

#### 3.1.1. Effects of Test-Section Length on Critical Swimming Speed

**Figure 2:**
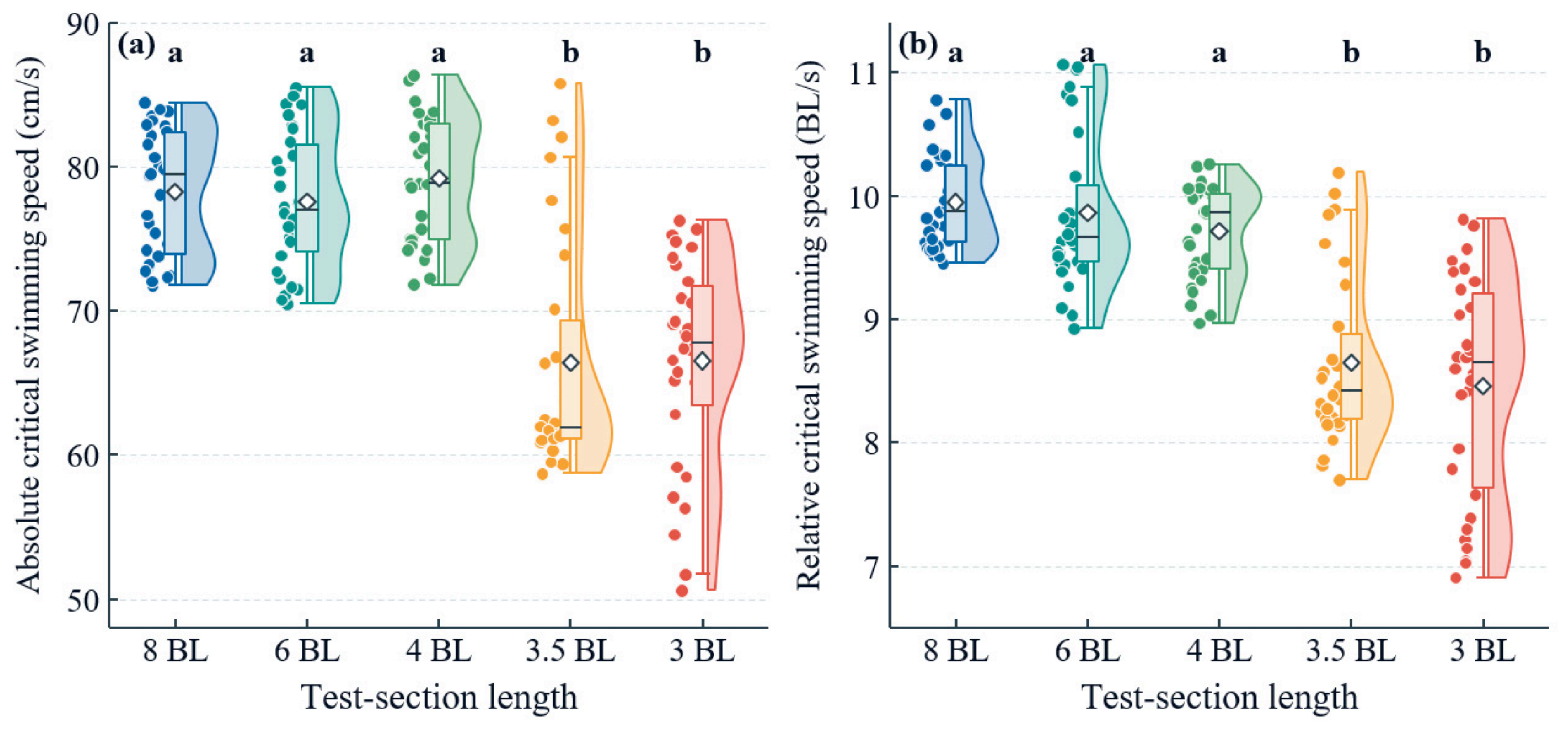
Critical swimming speed of juvenile grass carp with a mean BL of 7.92 ± 0.61 cm under different test-section lengths: (**a**) absolute critical swimming speed; (**b**) relative critical swimming speed. Colored points represent measurements from individual fish; violin widths indicate kernel density distributions; the lower and upper bounds of each box represent the 25th and 75th percentiles, respectively; the horizontal line within the box indicates the median; and the whiskers extend to the most extreme values within 1.5 times the interquartile range; diamonds indicate the means. Different lowercase letters indicate significant differences among treatments (*p* < 0.05).

Within the length experiment using juvenile grass carp with a mean BL of 7.92 ± 0.61 cm, critical swimming performance showed a threshold-like pattern across the tested section lengths (Figure 2). When the test-section lengths were 8 BL, 6 BL, and 4 BL, mean absolute critical swimming speeds were 78.27, 77.59, and 79.18 cm/s, respectively, and mean relative critical swimming speeds were 9.95, 9.86, and 9.71 BL/s, respectively. The similar mean values indicate that critical swimming performance remained relatively stable when the test-section length was not less than 4 BL. As the test-section length was further shortened to 3.5 BL and 3 BL, mean absolute critical swimming speed decreased to 66.37 and 66.51 cm/s, respectively, and mean relative critical swimming speed decreased to 8.65 and 8.45 BL/s, respectively. Compared with the 4 BL condition, the 3.5 BL condition reduced absolute critical swimming speed by 12.82 cm/s (95% BCa CI: 9.14–15.71 cm/s), a decrease of 16.18%, and reduced relative critical swimming speed by 1.06 BL/s (95% BCa CI: 0.76–1.32 BL/s), a decrease of 10.96%. The dispersion of critical swimming-speed data also increased markedly under the 3.5 BL and 3 BL conditions. Kruskal–Wallis tests indicated overall differences among test-section-length conditions in absolute critical swimming speed (*H*(4) = 68.45, *p* < 0.001, *ε*^2^ = 0.459) and relative critical swimming speed (*H*(4) = 74.09, *p* < 0.001, *ε*^2^ = 0.497). Dunn–Bonferroni comparisons showed no differences among the 8, 6, and 4 BL conditions or between the 3.5 and 3 BL conditions, whereas the 3.5 and 3 BL conditions had lower critical swimming speeds than the three longer test-section conditions.

#### 3.1.2. Effects of Test-Section Length on Tail-Beat Motion and Burst Behavior

**Figure 3:**
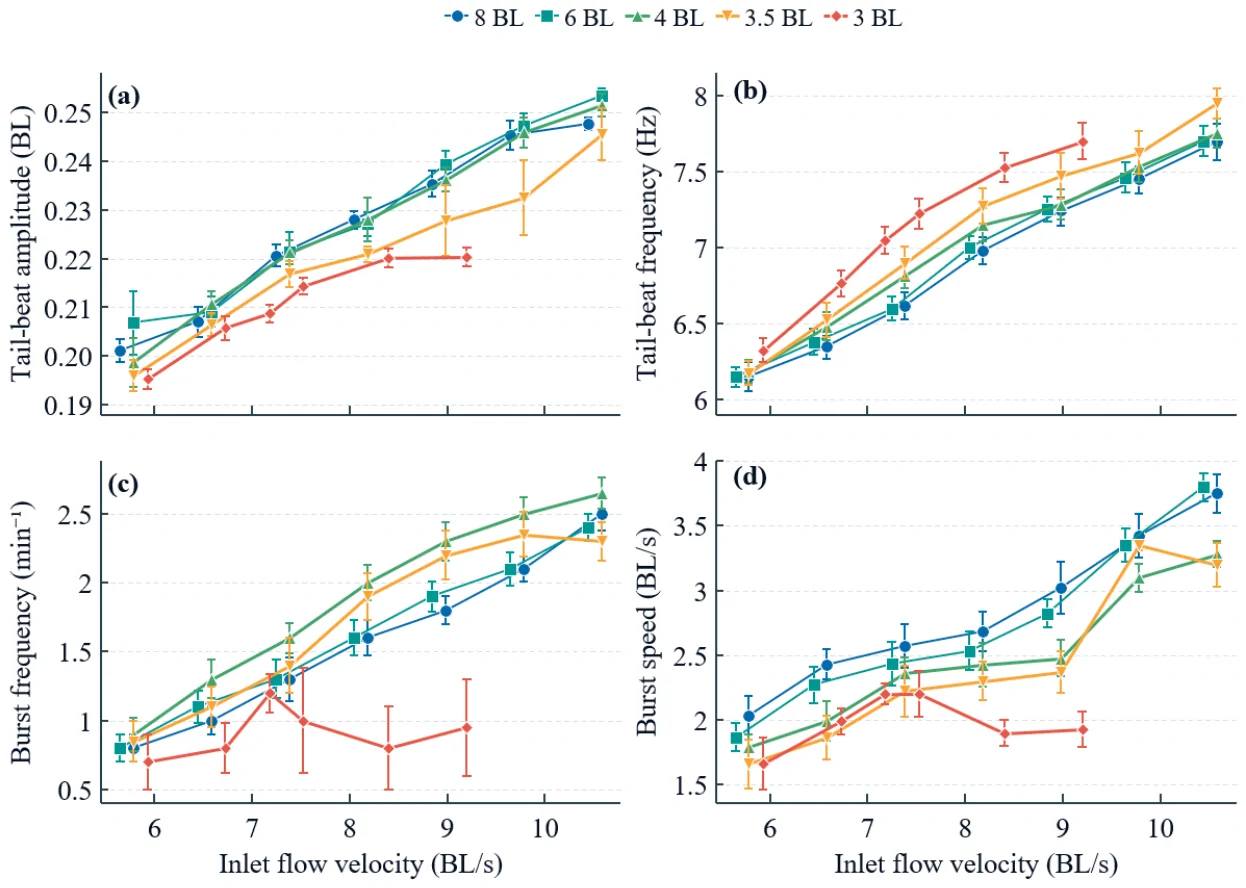
Tail-beat motion and burst behavior of juvenile grass carp under different test-section lengths: (**a**) tail-beat amplitude; (**b**) tail-beat frequency; (**c**) burst frequency; (**d**) burst speed. Symbols and lines represent means, and error bars indicate standard deviations.

Tail-beat amplitude and tail-beat frequency generally increased with increasing flow velocity under all conditions, but the adjustment patterns differed among test-section lengths (Figure 3a,b). Under the 8 BL and 6 BL conditions, tail-beat parameters changed relatively smoothly with flow velocity. For example, under the 8 BL condition, tail-beat amplitude increased from approximately 0.20 to 0.25 BL, tail-beat frequency increased from 6.15 to 7.70 Hz, and data dispersion remained relatively small. Under the 4 BL condition, both tail-beat amplitude and frequency increased with flow velocity at low and intermediate velocities; however, when velocity exceeded 8.00 BL/s, the increase in tail-beat amplitude slowed, and the fluctuation range of tail-beat frequency increased. When the test section was shortened to 3.5 BL and 3 BL, the pattern of tail-beat parameters changed. Under the 3.5 BL condition, tail-beat amplitude increased from approximately 0.20 BL to nearly 0.25 BL, but was generally lower than that under the 4–8 BL conditions at some intermediate and high velocities, with relatively greater data dispersion. Under the 3 BL condition, tail-beat amplitude increased from approximately 0.19 to 0.22 BL and then tended to stabilize, whereas tail-beat frequency increased from 6.33 to 7.70 Hz. Overall, within the length gradient used in this study, shortening the test section from 4 BL to 3.5 BL or less was accompanied by smaller increases in tail-beat amplitude and greater reliance on increased tail-beat frequency at higher flow velocities.

Burst frequency of juvenile grass carp generally increased with increasing flow velocity under all conditions. Except for the 3 BL condition, where burst speed decreased at intermediate and high velocities, burst speed generally increased under the other conditions, as shown in Figure 3c,d. Under the 8 BL and 6 BL conditions, burst behavior changed relatively continuously with flow velocity. For example, under the 8 BL condition, burst frequency increased from 0.80 to 2.50 events/min, and burst speed increased from 2.03 to 3.75 BL/s. Under the 4 BL condition, burst frequency increased from 0.90 to 2.65 events/min, and burst speed increased from 1.79 to 3.28 BL/s. However, when velocity exceeded 8.00 BL/s, the increase in burst speed slowed, and the range of data fluctuation increased, indicating that longitudinal burst adjustment at high velocities may have begun to be constrained at this length. Under the 3.5 BL condition, burst frequency increased from 0.85 to 2.30 events/min, and burst speed fluctuated upward from 1.66 to 3.20 BL/s, with relatively high overall dispersion. Under the 3 BL condition, burst frequency fluctuated markedly with flow velocity, and burst speed reached approximately 2.20 BL/s at intermediate velocities before decreasing to 1.89–1.92 BL/s at high velocities. These results indicate that burst behavior became less stable when the test-section length was less than 4 BL. In particular, under the 3 BL condition, burst speed no longer increased at high flow velocities, and longitudinal position adjustment was reduced.

#### 3.1.3. Effects of Test-Section Length on Swimming-State Distribution

**Figure 4:**
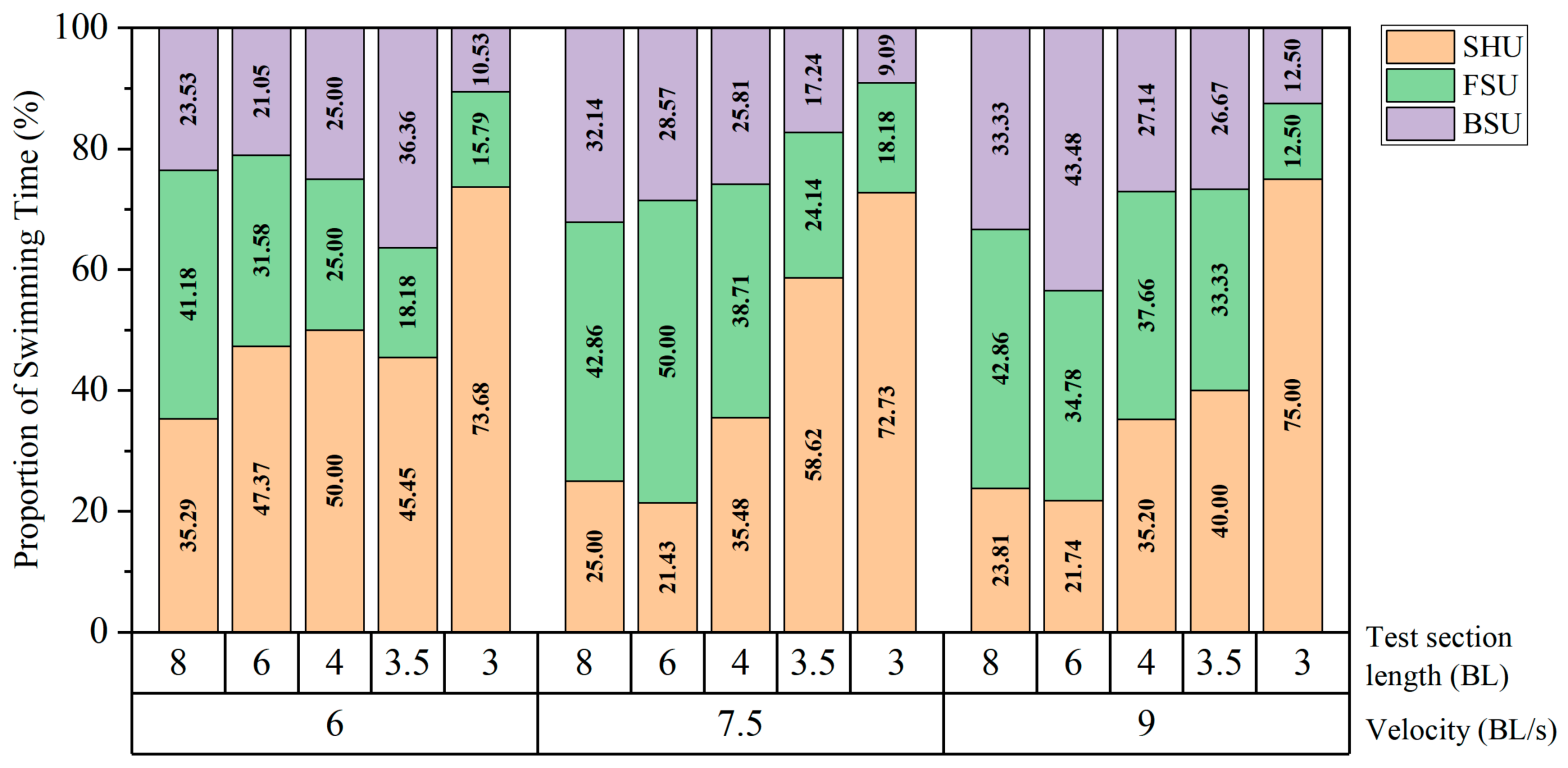
Time budget of swimming states of juvenile grass carp under different test-section lengths and inlet velocities.

Analysis of the time proportions of swimming states under different flow velocities and test-section lengths showed that spatial constraint had a nonlinear effect on behavioral strategy (Figure 4). During the formal testing stage included in the statistics, passive drifting downstream was not observed under any test-section length condition. Within the relatively ample space of 8 BL to 4 BL, juvenile grass carp displayed relatively stable and coherent locomotor strategies. For example, under the 4 BL condition, as flow velocity increased from 6.0 to 9.0 BL/s, the proportion of station holding upstream (SHU) decreased from 50.00% to 35.06%, whereas the proportion of forward swimming upstream (FSU) increased from 25.00% to 37.66%, reflecting more active resistance to the flow at high velocity. However, once the test space was compressed below 4 BL (3.5 BL and 3 BL), the swimming-state distributions changed markedly at each velocity. Under the 3 BL condition, juvenile grass carp showed substantially less longitudinal displacement and spent a greater proportion of time holding station. At flow velocities of 6.0, 7.5, and 9.0 BL/s, the SHU proportion increased sharply to more than 72.00%, whereas FSU and upstream-facing backward movement (BSU) were severely suppressed. Overall, for juvenile grass carp with a mean BL of 7.92 ± 0.61 cm, the behavioral changes observed between the 4 and 3.5 BL conditions were consistent with a threshold-like pattern within the tested length range. Test-section lengths below 4 BL were associated with more restricted displacement and altered swimming-state distributions under the present experimental conditions.

### 3.2. Effects of Cross-Sectional Blockage Ratio on Critical Swimming Speed and Swimming Behavior of Juvenile Grass Carp

#### 3.2.1. Effects of Cross-Sectional Blockage Ratio on Critical Swimming Speed

**Figure 5:**
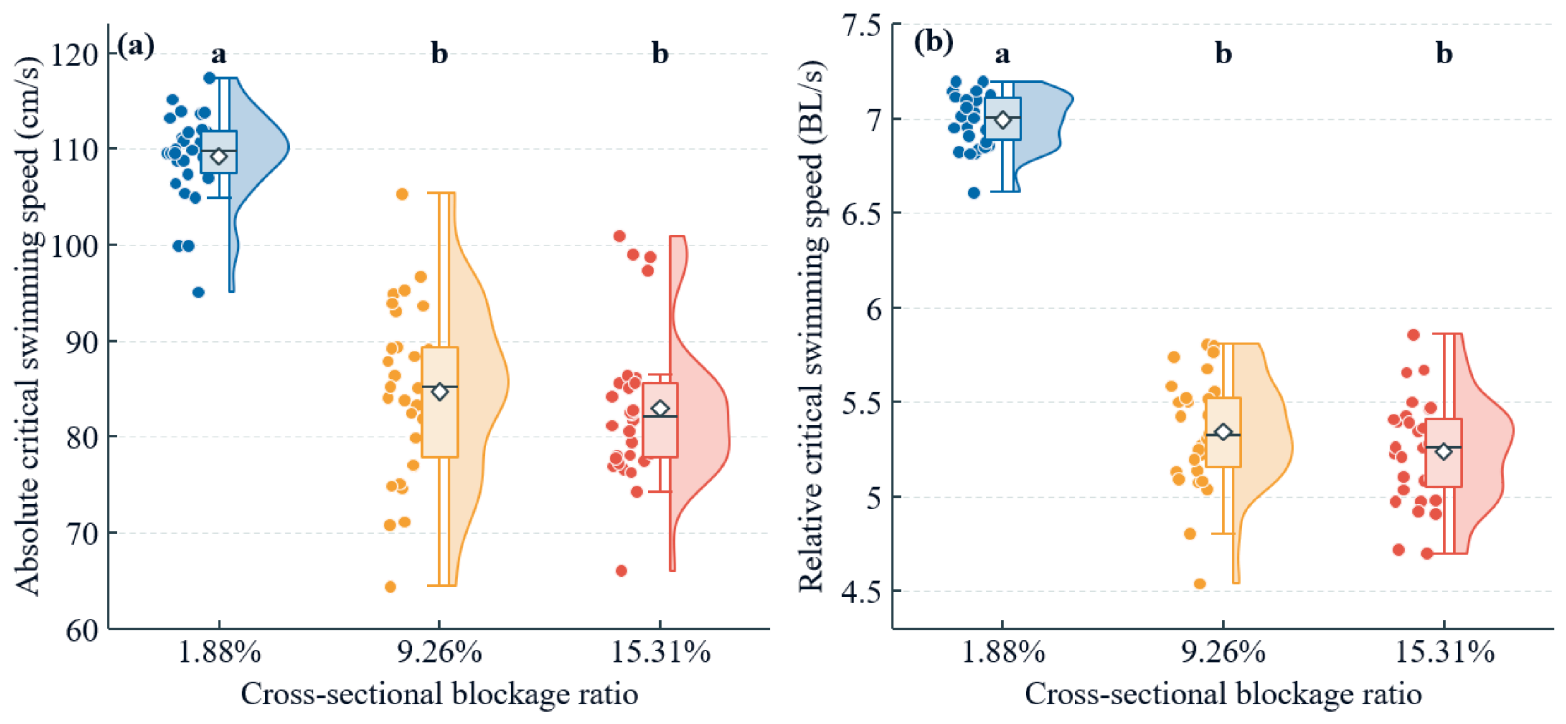
Critical swimming speed of juvenile grass carp with a mean BL of 15.76 ± 0.97 cm under different cross-sectional blockage ratios: (**a**) absolute critical swimming speed; (**b**) relative critical swimming speed. Different lowercase letters indicate significant differences (*p* < 0.05).

For juvenile grass carp with a mean BL of 15.76 ± 0.97 cm, absolute and relative critical swimming speeds decreased markedly when the cross-sectional blockage ratio increased from 1.88% to 9.26%. After the blockage ratio further increased to 15.31%, the mean values decreased only slightly and did not differ significantly from those under the 9.26% condition (Figure 5). Under the 1.88% blockage-ratio condition, juvenile grass carp showed the strongest swimming performance, with a mean absolute critical swimming speed of 109.24 ± 4.72 cm/s and a mean relative critical swimming speed of 6.99 ± 0.14 BL/s. When the blockage ratio increased to approximately 10%, boundary constraint and local flow-field distortion within the channel suppressed swimming performance: mean absolute critical swimming speed decreased to 84.68 ± 8.99 cm/s, corresponding to a reduction of 24.56 cm/s (95% BCa CI: 20.98–28.18 cm/s) or 22.48% relative to the 1.88% group, while mean relative critical swimming speed decreased to 5.34 ± 0.29 BL/s, corresponding to a reduction of 1.65 BL/s (95% BCa CI: 1.54–1.77 BL/s) or 23.60%. When the blockage ratio further increased to approximately 15%, the constraints imposed by physical space and hydrodynamic resistance on juvenile fish movement reached their maximum. Mean absolute critical swimming speed decreased further to 82.92 ± 7.77 cm/s, a cumulative 24.09% decrease relative to the 1.88% group, and mean relative critical swimming speed decreased to 5.24 ± 0.27 BL/s, a cumulative 25.10% decrease. Kruskal–Wallis tests indicated overall differences among blockage-ratio conditions in absolute critical swimming speed (*H*(2) = 58.27, *p* < 0.001, *ε*^2^ = 0.655) and relative critical swimming speed (*H*(2) = 60.44, *p* < 0.001, *ε*^2^ = 0.679). Dunn–Bonferroni comparisons showed that the 1.88% group had higher absolute and relative critical swimming speeds than the 9.26% and 15.31% groups, whereas no difference was detected between the 9.26% and 15.31% groups.

#### 3.2.2. Effects of Cross-Sectional Blockage Ratio on Tail-Beat Motion and Burst Behavior

**Figure 6:**
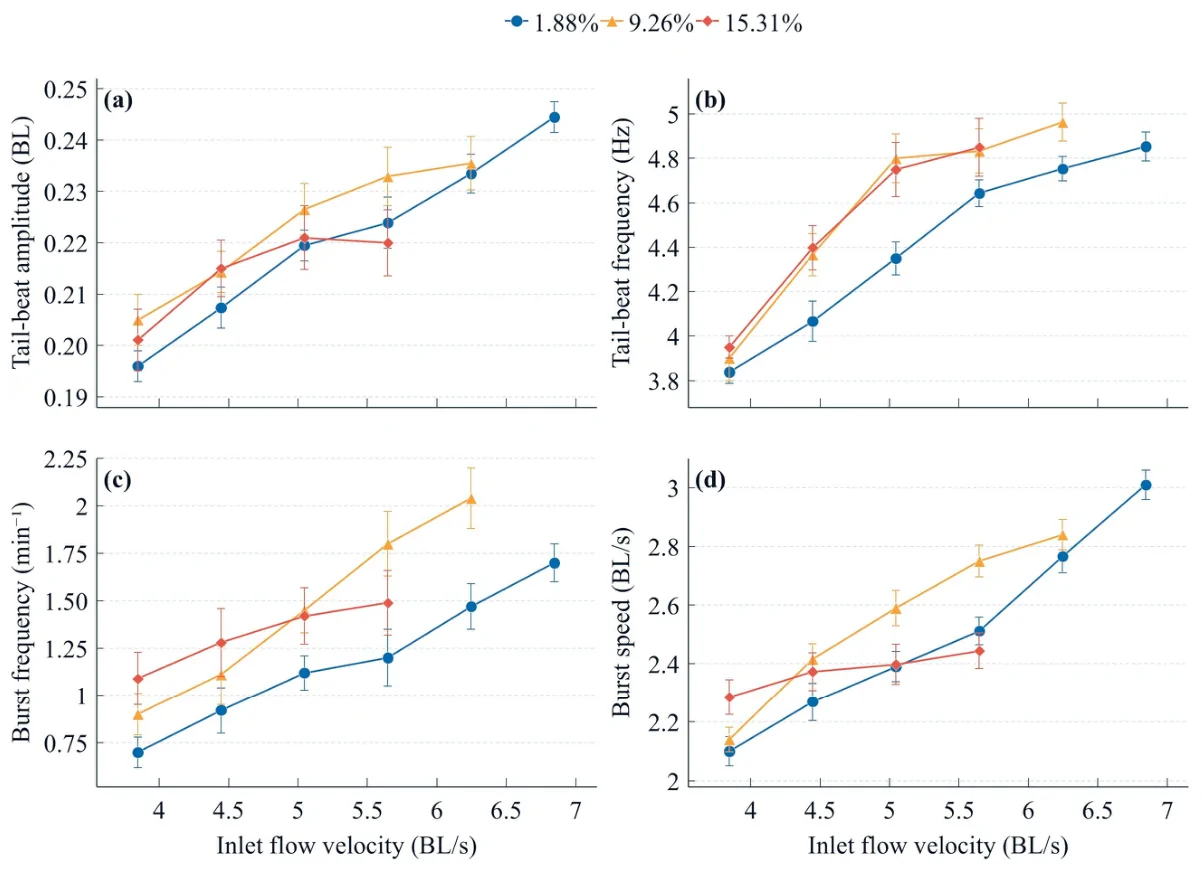
Tail-beat motion and burst behavior of juvenile grass carp under different cross-sectional blockage ratios: (**a**) tail-beat amplitude; (**b**) tail-beat frequency; (**c**) burst frequency; (**d**) burst speed. Symbols and lines represent means, and error bars indicate standard deviations.

With increasing inlet velocity, tail-beat amplitude and tail-beat frequency of juvenile grass carp generally increased, but their variation patterns differed among cross-sectional blockage-ratio conditions (Figure 6a,b). Under the 1.88% cross-sectional blockage-ratio condition, tail-beat amplitude increased from 0.196 to 0.244 BL, an increase of 24.74%, and tail-beat frequency increased from 3.84 to 4.85 Hz. Overall changes were relatively smooth, and data dispersion was relatively small, indicating that fish could respond to increased flow velocity by simultaneously increasing tail-beat amplitude and tail-beat frequency. In contrast, under higher cross-sectional blockage ratios (9.26% and 15.31%), tail-beat frequency was generally higher in the intermediate- and high-velocity ranges; data dispersion also increased, and the increase in tail-beat amplitude was relatively restricted. These results indicate that, as cross-sectional blockage ratio increases, the increase in lateral tail-beat amplitude became more limited, whereas behavioral adjustment relied more on tail-beat frequency and inter-individual variability increased.

Within the corresponding test-velocity range of each condition, burst frequency and burst speed of juvenile grass carp generally increased with flow velocity (Figure 6c,d). Under the low cross-sectional blockage-ratio condition (1.88%), burst frequency increased from 0.70 to 1.70 events/min, an increase of 142.9%, and burst speed increased from 2.10 to 3.01 BL/s, an increase of 43.3%, with relatively small data dispersion. At a cross-sectional blockage ratio of 9.26%, the increases in burst frequency and burst speed were 126.7% and 32.7%, respectively. When the cross-sectional blockage ratio increased to 15.31%, burst frequency increased from 1.09 to 1.49 events/min, while burst speed increased only from 2.29 to 2.44 BL/s, and data dispersion increased. Overall, under high cross-sectional blockage-ratio conditions, the magnitude of change in burst speed was relatively small, suggesting that stronger spatial constraint may weaken the ability of fish to respond to velocity changes by increasing burst speed and may produce greater dispersion in individual behavioral responses.

#### 3.2.3. Effects of Cross-Sectional Blockage Ratio on Swimming-State Distribution

**Figure 7:**
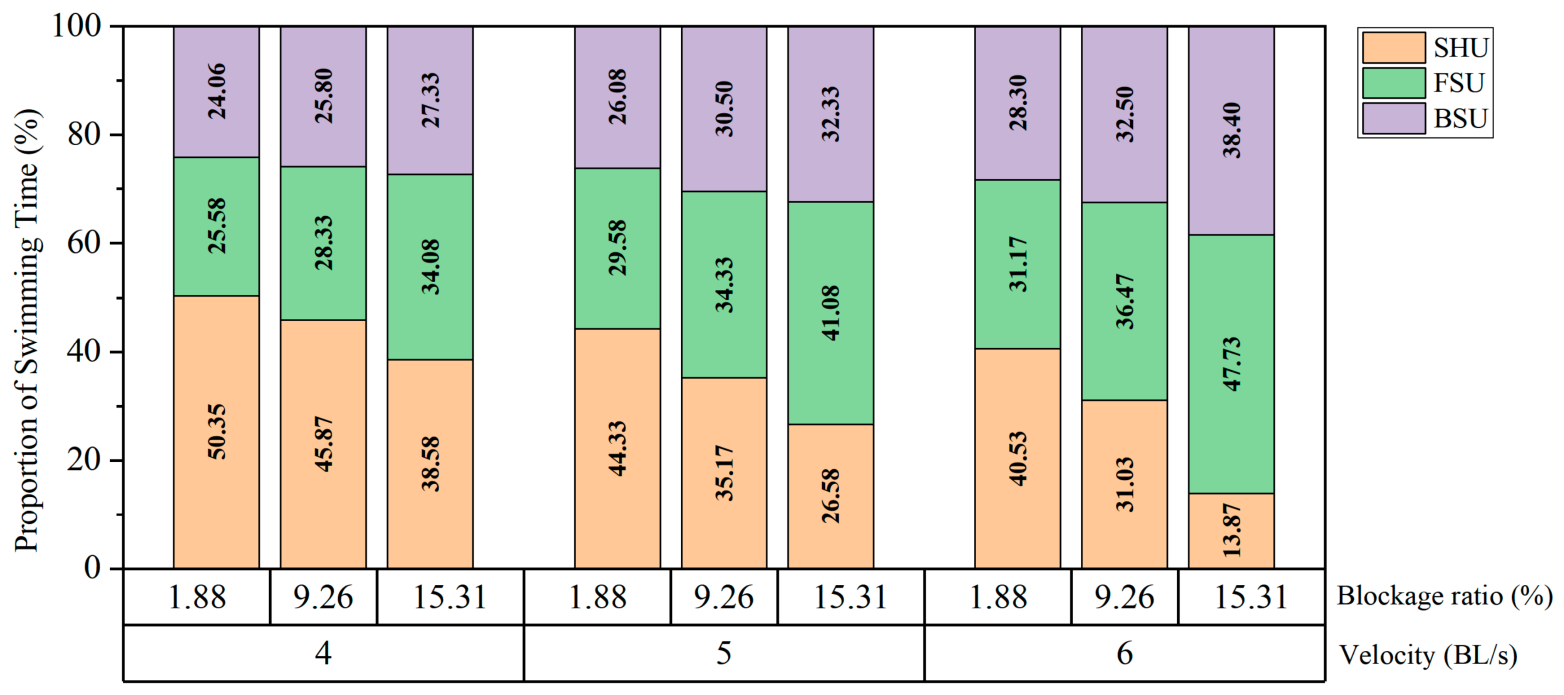
Time budget of swimming states of juvenile grass carp under different cross-sectional blockage ratios and inlet velocities.

The time budget of swimming states of juvenile grass carp under different cross-sectional blockage ratios and inlet velocities is shown in Figure 7. During the formal testing stage under all cross-sectional blockage-ratio conditions, passive drifting downstream was not observed. As velocity increased from 4 to 6 BL/s, test fish under all conditions showed a decrease in the proportion of station holding upstream (SHU) and increases in the proportions of forward swimming upstream (FSU) and upstream-facing backward movement (BSU). However, cross-group comparisons at specific velocities showed that increasing blockage ratio disrupted the movement strategy of juvenile fish. At the low velocity of 4 BL/s, differences among groups were small, and fish in all groups maintained steady swimming with relatively high SHU proportions (38.58–50.35%). When velocity increased to 5 BL/s, the spatial compression effect became evident. Under the 1.88% blockage-ratio condition, the SHU proportion remained 44.33%, whereas under the 9.26% and 15.31% blockage ratios, narrowing of the available space forced fish to alternate between bursts and downstream retreat, reducing SHU proportions to 35.17% and 26.58%, respectively. At the high velocity of 6 BL/s, this cross-group deterioration reached its maximum. Under the 1.88% blockage-ratio condition, fish could still regulate movement autonomously with SHU and FSU proportions of 40.53% and 31.17%, respectively. In contrast, under the 15.31% blockage-ratio condition, the SHU proportion dropped sharply to 13.87%, while the proportions of FSU (47.73%) and BSU (38.40%) reached their highest values among all groups, indicating less stable longitudinal station holding and more frequent alternation between upstream bursts and downstream retreat. These comparisons indicate that, under the same nominal test-section velocity, higher blockage ratios were associated with less stable station holding and more frequent alternation between upstream bursts and downstream retreat. These behavioral changes were consistent with the observed reduction in critical swimming speed under higher blockage conditions.

### 3.3. Flow Velocity Distribution

**Figure 8:**
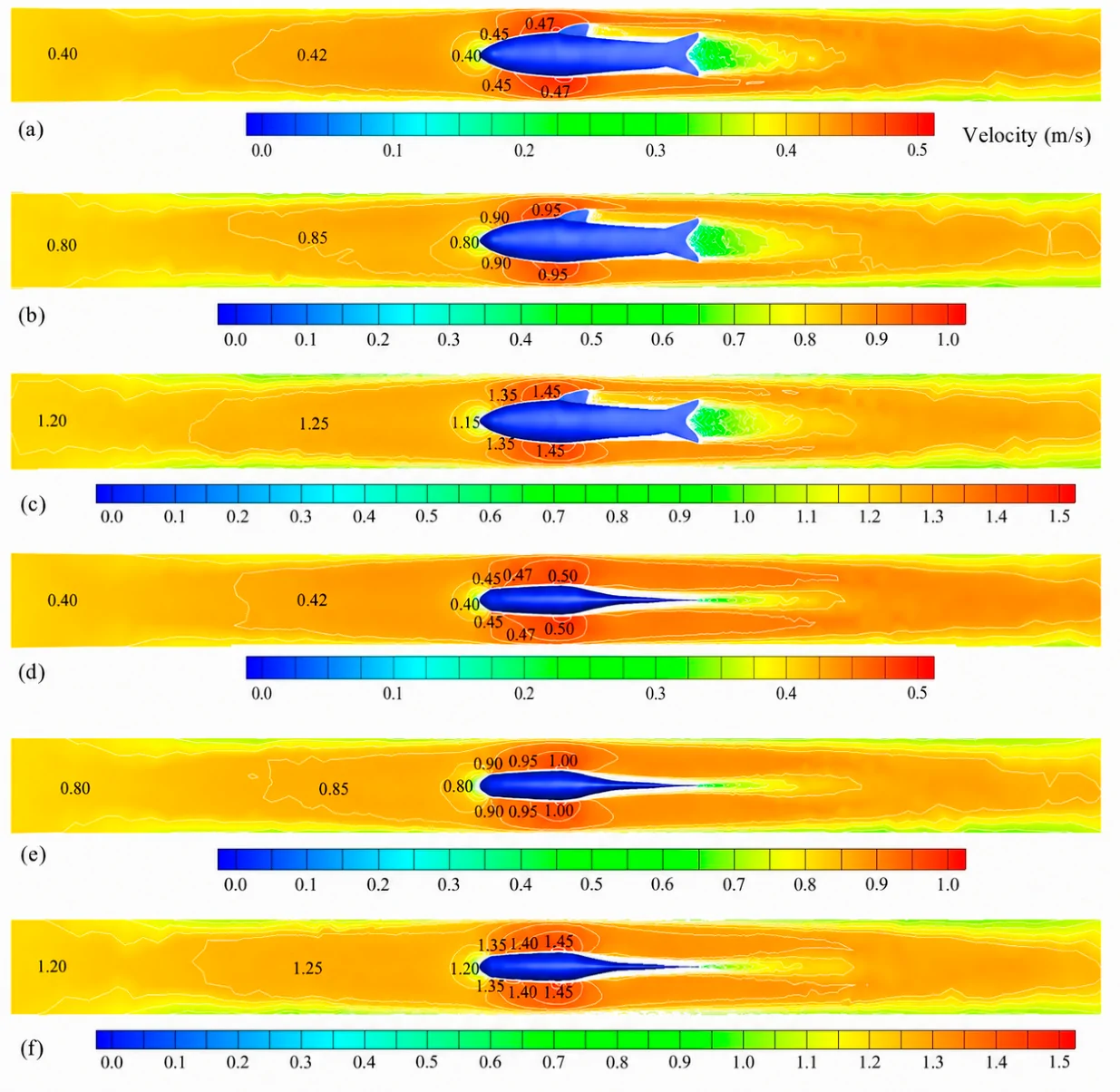
Streamwise velocity distribution when the simulated fish was located at the center of the test section under an approximately 10% (9.26%) cross-sectional blockage ratio: (**a**–**c**) longitudinal vertical center plane at inlet velocities of 0.4, 0.8, and 1.2 m/s, respectively; (**d**–**f**) longitudinal horizontal center plane at inlet velocities of 0.4, 0.8, and 1.2 m/s, respectively.

When juvenile grass carp were positioned at the lateral and vertical center of the test section, the surrounding flow field showed an overall symmetrical distribution. Incoming flow separated in front of the fish head, bypassed the fish body, and formed local high-velocity regions along both sides and above and below the maximum cross-section, while a low-velocity wake region formed downstream of the fish. As inlet velocity increased from 0.4 to 0.8 and 1.2 m/s, streamwise velocity in the local high-velocity regions increased accordingly, but the magnitude of increase differed among locations, indicating clear spatial non-uniformity in the actual flow field around the fish body (Figure 8).

At inlet velocities of 0.4, 0.8, and 1.2 m/s, the maximum streamwise velocities near the maximum cross-section of the fish were approximately 0.50, 1.00, and 1.45 m/s, respectively, representing increases of 25.0%, 25.0%, and 20.8% over the corresponding inlet velocities. The 0.8 m/s inflow was close to the measured critical swimming speed of 0.847 m/s under the 9.26% blockage-ratio condition. At this inflow, the local streamwise velocity near the maximum cross-section of the fish had reached approximately 1.00 m/s, indicating that the local hydrodynamic conditions actually experienced by the test fish may have been markedly stronger than the overall level represented by the nominal inlet velocity.

These results show that when the blockage ratio approaches 10%, even if the fish remains at the center of the test section, occupation of the flow cross-section by the fish body concentrates the flow into the remaining surrounding space and produces pronounced bypass-flow acceleration near the maximum cross-section of the fish. Therefore, a high blockage ratio not only compresses the space available for tail beating and postural adjustment, but also increases the spatial non-uniformity of local velocity around the fish body. This provides a hydrodynamic explanation for the decrease in critical swimming speed, the reduction in stable station-holding time, and the increase in tail-beat frequency near a 10% blockage ratio.

## 4. Discussion

### 4.1. Effects of Test-Section Spatial Scale on Critical Swimming Speed

This study shows that the spatial scale of the test section in a closed flume systematically affects critical swimming speed and swimming behavior in juvenile grass carp. Sustained forced swimming during the 15-min velocity steps may have produced progressive physiological fatigue, which formed part of the physiological basis of the measured critical swimming performance. In the test-section length experiment, juvenile grass carp with a mean body length of 7.92 cm had absolute critical swimming speeds of 0.664–0.792 m/s and relative critical swimming speeds of 8.45–9.95 BL/s, which fall within the ranges reported by Tan et al. [33] for juvenile grass carp. The numerical comparison should nevertheless be interpreted in light of differences in fish size and testing protocol: Tan et al. tested grass carp ranging from 2 to 8 cm using 20-min velocity steps and a 5-s exhaustion criterion, whereas the present study used fish with a narrower size distribution, 15-min formal velocity steps, and a continuous 20-s screen-impingement criterion. More importantly, Tan et al. [33] primarily examined body-size and interspecific differences under a single flume configuration, while Li et al. [26] focused on body-length-related variation in larger common carp tested in an open-type flume. Tudorache et al. [34] demonstrated that flume length affected critical swimming speed and burst–glide behavior in common carp, but their experiment mainly compared long and short flume conditions. Building on these studies, the present experiment maintained a constant cross-section with a low blockage ratio and examined five relative test-section lengths from 3 to 8 BL in a single size class of juvenile grass carp. Critical swimming speed remained relatively stable under the 4, 6, and 8 BL conditions but decreased significantly under the 3.5 and 3 BL conditions, revealing a threshold-like pattern between the tested 4 and 3.5 BL conditions. The accompanying changes in longitudinal displacement, tail-beat regulation, and burst behavior further provide behavioral evidence for how insufficient longitudinal space affects measured swimming performance. Thus, the present study extends previous evidence that flume length influences swimming performance by narrowing the scale range over which this effect emerged and linking the change in critical swimming speed to specific locomotor adjustments. Ashraf et al. [35] found that in-flume habituation time and behavior during the habituation stage affect measured swimming performance. A synthesis of 167 studies by Jeremy et al. [36] also showed that swimming speeds obtained from forced swimming tests in closed flumes are usually lower than those obtained from volitional swimming tests, and that spatial limitations of the apparatus may prevent fish from expressing different swimming gaits. The incremental-velocity protocol, fatigue criterion, and critical swimming-speed calculation used in this study are consistent with NB/T 10612-2021, Specification for Swimming Ability Test of Target Fishes for Hydropower Projects [24]. On this basis, the present study provides additional quantitative evidence for the influence of effective test-section length and cross-sectional scale on test results.

The effect of cross-sectional blockage ratio on critical swimming speed cannot be eliminated solely by conventional solid-blockage correction. The Bell–Terhune calculation described in Section 2.6 yielded estimated solid-blockage coefficients of 0.67%, 7.00%, and 14.69% for the 1.88%, 9.26%, and 15.31% conditions, respectively. The corresponding adjusted absolute *U*_crit_ values were 109.97, 90.60, and 95.10 cm/s, while the adjusted relative *U*_crit_ values were 7.04, 5.71, and 6.01 BL/s, respectively. These values provide a first-order estimate of the nominal solid-blockage component. Although the Bell–Terhune equation has been conventionally applied in fish swimming tunnels [31,32], it represents the fish body as a fixed streamlined obstruction and does not account for body deformation, active thrust generation, tail-beat-induced flow unsteadiness, changes in swimming position, or non-uniform wall interactions. Kline et al. [32] also found that the standard Bell–Terhune equation underestimated the solid-blockage effect measured near the fish body and that its performance depended on the fish-shape coefficient and area exponent. Consistent with this limitation, the numerical results of the present study showed that, under the 9.26% condition, local streamwise velocity near the maximum fish cross-section reached 1.21–1.25 times the inlet velocity and was spatially non-uniform. Such local variation cannot be represented fully by a single correction coefficient. Thus, conventional solid-blockage correction alone cannot fully characterize the blockage effects observed in the present study.

### 4.2. Swimming-Behavior Responses and Mechanisms Under Spatial Constraint

A major contribution of this study is that it links changes in critical swimming speed with tail-beat motion, burst behavior, and swimming states, thereby providing behavioral evidence for the responses observed under longitudinal and cross-sectional spatial constraints. Bainbridge [27] and Webb et al. [28] showed that fish increase swimming speed by coordinately increasing tail-beat amplitude and tail-beat frequency, whereas Videler and Weihs [37] proposed that burst-and-coast swimming helps reduce energy costs during high-speed swimming. In the present study, when test-section length was 4–8 BL, juvenile grass carp could coordinately increase tail-beat amplitude, tail-beat frequency, and burst speed as flow velocity increased, and critical swimming speed remained relatively stable. When the test section was shorter than 4 BL, increases in tail-beat amplitude and burst speed were suppressed, while tail-beat frequency and its fluctuation increased. This indicates that fish needed to maintain position through high-frequency tail beating, but longitudinal space was insufficient to support full acceleration, gliding, and renewed bursts after downstream retreat. Such spatially constrained locomotor regulation may also have reduced opportunities for energy-saving burst-and-coast swimming, thereby increasing energy expenditure and accelerating fatigue during the 15-min velocity steps.

Cross-sectional compression was associated with both local flow modification and constraints on the space available for movement. At 6 BL/s, as the blockage ratio increased from 1.88% to 15.31%, the proportion of station holding upstream decreased from 40.53% to 13.87%, while the proportions of forward swimming upstream and upstream-facing backward movement increased to 47.73% and 38.40%, respectively. These changes were accompanied by restricted growth in tail-beat amplitude, increased tail-beat frequency, and more frequent burst-retreat behavior. The decrease in the SHU proportion indicates a weakened ability to maintain a stable spatial position, while the increase in BSU indicates that fish could still maintain an upstream-facing posture but could not fully offset the flow and therefore had to recover position through subsequent upstream bursts. Because endpoint definitions can influence measured swimming performance [14], the continuous 20-s screen-impingement criterion used here represents an operational endpoint of the *U*_crit_ test, rather than a direct measure of complete physiological exhaustion. The measured *U*_crit_ therefore indicates when fish could no longer maintain swimming against the flow under the specified flume conditions. Although the physiological and spatial contributions could not be quantified separately, all treatments followed the same protocol and termination criterion, and the reductions in *U*_crit_ coincided with changes in tail-beat motion, burst–retreat behavior, and local flow conditions, supporting an effect of spatial constraint on measured swimming performance. PDD denotes downstream movement accompanied by loss of upstream orientation, whereas grass carp may retreat downstream while maintaining positive rheotaxis [38]. Therefore, the absence of PDD should be interpreted within its behavioral definition and provides limited information about the physiological state at the *U*_crit_ endpoint. Consistently, a previous study using the same 20-s criterion reported downstream swimming only during the initial stage, but not during later high-velocity stages, in juvenile grass carp tested in 5-L and 50-L closed flumes [39].

From a hydrodynamic perspective, increased blockage ratio reduces the effective flow area between the fish body and the sidewalls, accelerates local flow around the fish, and may enhance velocity gradients and shear in narrow regions [31,32]. Zhang et al. [40] found that cross-sectional mean velocity in a vertical-slot fishway affected passage efficiency of juvenile grass carp, whereas Reynolds shear stress was closely related to path selection; fish that successfully ascended also actively used relatively low-velocity regions. These findings indicate that fish behavior depends not only on nominal velocity, but also on local velocity and shear conditions. The numerical results of the present study show that local bypass-flow acceleration around the fish body exposes different parts of the fish to non-uniform hydrodynamic forces. Together with the observed reductions in critical swimming speed and stable station holding, these findings suggest that the increased hydrodynamic resistance under stronger boundary constraint may increase the load required for fish to maintain posture and position. When the space for tail beating and postural adjustment is simultaneously compressed, such local flow-field changes may further weaken stable station-holding capacity and cause test fish to adopt high-frequency tail beating and repeated burst-retreat as an unsteady locomotor strategy. As cross-sectional space decreased, the observed restriction of lateral tail-beat amplitude was accompanied by greater reliance on high-frequency tail beating and repeated bursts. Di Santo and Goerig [41] noted that fish can use boundaries and flow-field heterogeneity to adjust locomotor strategies and reduce locomotor costs. Therefore, the reduction in critical swimming speed under high blockage ratios should not be attributed simply to increased drag on the fish body, but should be understood as the combined result of local bypass-flow changes, near-wall effects, and movement-space compression. By comparison, shortened test-section length mainly restricts longitudinal acceleration, gliding, and position recovery, whereas increased blockage ratio not only restricts tail beating and postural adjustment but also alters local flow conditions around the fish body. Although 10% is commonly recommended as the threshold above which blockage effects should be considered [24,32], the pronounced decline in critical swimming speed at 9.26% suggests that a more stringent criterion may be appropriate for juvenile grass carp. Accordingly, a blockage ratio below 5% is proposed as a precautionary recommendation based on the observed trend and previous fish swimming studies that maintained blockage below 5% to minimize flow interference [42,43], with its precise applicability requiring further evaluation.

Test-section length was expressed relative to body length, and cross-sectional spatial constraint was characterized using blockage ratio. The test-section-length and blockage-ratio experiments used juvenile grass carp with mean BLs of approximately 7.92 and 15.76 cm, respectively. Within each experiment, fish from the same size group were allocated sequentially among treatments without deliberate selection, and treatment effects were evaluated through comparisons among the corresponding experimental conditions. The resulting test-section-length and blockage-ratio criteria apply primarily to juvenile grass carp within their respective experimental size ranges and to fish with similar morphology and propulsion modes. For individuals substantially outside these size ranges, the criteria should be further evaluated in relation to body morphology and actual blockage conditions.

Future research could further expand the range of fish species, body sizes, and life-history stages examined to compare responses to longitudinal and cross-sectional spatial constraints among fishes with different morphologies and propulsion modes, and to verify the applicability of the scale recommendations obtained in this study. Comparative experiments should also be conducted in closed flumes and open-channel flumes of different scales to clarify the effects of the free surface and boundary conditions on swimming behavior and performance parameters. Integrating three-dimensional behavioral tracking, particle image velocimetry, and numerical simulation would further reveal the coupling among fish position, locomotor posture, tail-beat motion, and the local instantaneous flow field, and would help improve spatial-constraint evaluation and blockage-correction methods for different fish species and test apparatus. Such work would improve the accuracy and cross-system comparability of swimming-performance data and provide more reliable biological evidence for hydraulic design and operational optimization of fish passage facilities.

## 5. Conclusions

Using two independent experiments conducted with different size classes of juvenile grass carp, this study examined the effects of test-section length and cross-sectional blockage ratio on measured swimming performance, behavioral responses, and local hydrodynamic conditions. The main conclusions are as follows.

(1) Critical swimming speed showed a threshold-like pattern across the tested section lengths. When the test-section lengths were 8, 6, and 4 BL, absolute critical swimming speeds were 77.59–79.18 cm/s and relative critical swimming speeds were 9.71–9.95 BL/s, with no significant differences among groups (*p* > 0.05). When the length was shortened to 3.5 and 3 BL, critical swimming speed decreased significantly (*p* < 0.001). Compared with the 4 BL condition, the 3.5 BL condition reduced absolute and relative critical swimming speeds by 16.18% and 10.96%, respectively. Under the present experimental conditions, juvenile grass carp approximately 8 cm in body length exhibited a threshold-like transition in critical swimming speed between the tested test-section lengths of 3.5 and 4 BL.
(2) Shortening the test section mainly altered locomotor regulation by limiting longitudinal displacement and positional recovery of juvenile grass carp. Under the 4–8 BL conditions, test fish could coordinately adjust tail-beat amplitude, tail-beat frequency, and burst behavior as flow velocity increased. When the length was less than 4 BL, increases in tail-beat amplitude and burst speed were restricted, whereas tail-beat frequency and its fluctuation increased. Under the 3 BL condition, burst speed decreased to 1.89–1.92 BL/s at high velocities, and the proportion of station holding upstream exceeded 72% at all tested velocities. These results indicate that an excessively short test section compresses the movement range available for forward swimming, downstream retreat, and re-establishing position, causing fish to rely more on high-frequency tail beating to maintain station.
(3) Increasing cross-sectional blockage ratio reduced the critical swimming speed of juvenile grass carp and altered the local hydrodynamic conditions around the fish body. In this study, cross-sectional blockage was characterized as the ratio of the maximum cross-sectional area of juvenile grass carp to the flow cross-sectional area of the test section. When the blockage ratio increased from 1.88% to 9.26%, absolute and relative critical swimming speeds decreased from 109.24 cm/s and 6.99 BL/s to 84.68 cm/s and 5.34 BL/s, representing reductions of 22.48% and 23.60%, respectively. When the blockage ratio increased to 15.31%, the cumulative reductions reached 24.09% and 25.10%, respectively. Numerical simulation showed that, under the 9.26% blockage ratio, local streamwise velocity near the maximum cross-section of the fish was 20.8–25.0% higher than the inlet velocity, indicating that the reduced effective flow area between the fish body and the sidewalls causes pronounced local bypass-flow acceleration and spatial non-uniformity in velocity.
(4) Cross-sectional compression was associated with both local bypass-flow acceleration and changes in the observed tail-beat kinematics and swimming-state allocation. As blockage ratio increased, the increase in tail-beat amplitude was suppressed, tail-beat frequency increased, and fish adjusted position more through bursts and downstream retreat. At 6 BL/s, the proportion of station holding upstream decreased from 40.53% under the 1.88% blockage-ratio condition to 13.87% under the 15.31% condition, while the proportions of forward swimming upstream and upstream-facing backward movement increased to 47.73% and 38.40%, respectively, indicating a marked weakening of stable station-holding capacity. For swimming-performance tests of juvenile grass carp similar in size to those used in the blockage-ratio experiment, a blockage ratio below 5% is recommended.

These findings can improve the reliability and cross-system comparability of fish swimming-performance data, thereby supporting biologically informed hydraulic design of fish-passage facilities and the ecological management of water infrastructure.

## Figures and Tables

**Table 1:** Morphological parameters of juvenile grass carp in the test-section length experiment.

Test-Section Length	8 BL	6 BL	4 BL	3.5 BL	3 BL	Total
Sample size (N)	30	30	30	30	30	150
Mean total length (cm)	9.82 ± 0.48	9.69 ± 0.69	9.95 ± 0.45	9.51 ± 0.61	9.71 ± 0.51	9.74 ± 0.57
Mean body length (cm)	8.02 ± 0.52	7.89 ± 0.71	8.19 ± 0.41	7.66 ± 0.64	7.88 ± 0.62	7.92 ± 0.61
Mean body weight (g)	8.32 ± 2.88	7.87 ± 3.33	9.36 ± 2.34	8.74 ± 2.96	8.58 ± 2.68	8.57 ± 2.86
Mean body depth (cm)	1.76 ± 0.26	1.74 ± 0.24	1.90 ± 0.30	1.80 ± 0.40	1.72 ± 0.38	1.78 ± 0.32
Body depth/body length	0.22 ± 0.03	0.22 ± 0.03	0.23 ± 0.05	0.23 ± 0.08	0.22 ± 0.04	0.23 ± 0.05
Mean body width (cm)	1.18 ± 0.22	1.14 ± 0.26	1.24 ± 0.36	1.19 ± 0.21	1.14 ± 0.26	1.18 ± 0.27
Body width/body length	0.15 ± 0.03	0.14 ± 0.02	0.15 ± 0.02	0.16 ± 0.02	0.14 ± 0.04	0.15 ± 0.03

**Table 2:** Morphological parameters of juvenile grass carp in the cross-sectional blockage-ratio experiment.

Cross-Sectional Blockage Ratio	1.88%	9.26%	15.31%	Total
Sample size (N)	30	30	30	90
Mean total length (cm)	18.43 ± 0.87	19.15 ± 1.15	19.19 ± 1.02	18.92 ± 1.07
Mean body length (cm)	15.63 ± 0.72	15.83 ± 1.17	15.82 ± 0.98	15.76 ± 0.97
Mean body weight (g)	61.22 ± 8.16	68.25 ± 12.65	68.95 ± 11.40	66.14 ± 11.34
Mean body depth (cm)	3.93 ± 0.27	3.85 ± 0.35	3.88 ± 0.31	3.89 ± 0.31
Body depth/body length	0.25 ± 0.02	0.24 ± 0.01	0.25 ± 0.01	0.25 ± 0.01
Mean body width (cm)	2.40 ± 0.15	2.55 ± 0.25	2.58 ± 0.22	2.51 ± 0.22
Body width/body length	0.15 ± 0.01	0.16 ± 0.01	0.16 ± 0.02	0.16 ± 0.01

## Data Availability

The data presented in this study are available from the corresponding authors upon reasonable request.

## Abbreviations

The following abbreviations are used in this manuscript:

BL	Body length
BSU	Upstream-facing backward movement
FSU	Forward swimming upstream
PDD	Passive drifting downstream
SHU	Station holding upstream
